# Prognostic value of stromal and epithelial periostin expression in human prostate cancer: correlation with clinical pathological features and the risk of biochemical relapse or death

**DOI:** 10.1186/1471-2407-12-625

**Published:** 2012-12-28

**Authors:** Pier Vitale Nuzzo, Alessandra Rubagotti, Linda Zinoli, Francesco Ricci, Sandra Salvi, Simona Boccardo, Francesco Boccardo

**Affiliations:** 1 IRCCS San Martino University Hospital – IST National Cancer Research Institute and the University of Genoa, Academic Unit of Medical Oncology (UOC Oncologia Medica B), Largo Rosanna Benzi 10, 16132 Genoa, Italy; 2 IRCCS San Martino University Hospital – IST National Cancer Research Institute, Pathology Unit, Genoa, Italy; 3 University of Genoa, Department of Internal Medicine, School of Medicine, Genoa, Italy

**Keywords:** POSTN protein, Human, Prostatic neoplasms, Extracellular matrix proteins, Prognosis, Biomarkers

## Abstract

**Background:**

The purpose of the present study was to evaluate the prognostic value of POSTN expression following prostatectomy.

**Methods:**

Periostin (POSTN) expression in prostate cancer (PCa) and in normal specimens was evaluated in 90 patients by an immuno-reactive score(IRS) based on the intensity of immunostaining and on the quantity of stained cells. The t-test was applied to compare IRS values in cancer specimens to values in normal specimens. Pearson’s test was used to correlate POSTN expression to clinical pathologic features. PSA progression-free and survival curves were constructed by the Kaplan–Meier method and compared using the log-rank test. Multi-parametric models were constructed according to the Cox technique adding all the covariates predicting for either PSA progression or death into the models after univariate analysis.

**Results:**

Both stromal and epithelial POSTN expression were significantly increased in tumor tissues**.** In particular, we found stromal expression to be significantly higher than epithelial expression as compared to normal tissues (p<0.000 and p=0.001).A significant correlation between POSTN epithelial expression and extra-prostatic extension was found (p=0.03). While high stromal expression was significantly associated with shorter survival (p=0.008), a low epithelial score significantly correlated with shorter PSA-free survival (p=0.04), suggesting that POSTN plays an apparently opposing biological role depending on its compartmentalization.Regardless of the mechanism that is involved, patients showing both high stromal and low epithelial expression made up a subgroup with a very bleak prognosis.

**Conclusions:**

Although requiring further validation through larger studies, our findings show that POSTN might represent a novel prognostic marker for PCa.

## Background

Prostate cancer (PCa) has become the most common malignancy among men in most Western countries [1]. Even when this tumor is apparently confined to the prostate, it encompasses a broad spectrum of diseases, some of which are characterized by extremely indolent behavior and others by very poor outcome. Therefore, an important clinical question is how aggressively to treat patients diagnosed at this stage. Among patients who are treated with radical prostatectomy, the most commonly used parameters for defining prognosis and choosing the right candidates for adjuvant local irradiation or systemic treatments include tumor volume and pathological grade and status of surgical margins, seminal vesicles and pelvic nodes [2]. However, there is no widely accepted method for quantifying tumor volume [3]**.** Moreover, tumor grade scoring methods can result in significant inter-observer variations, particularly when defining intermediate tumor grades [4,5]. This applies specifically to the old Gleason scoring method. New prognostic markers are therefore required.

Many of the cellular abnormalities that are present in most solid tumors are structural in nature and involve either the nuclear matrix (NM) or the extracellular matrix (ECM), both of which are regarded as a promising source of new markers [6-8]. Among the components of ECM, increasing interest has been shown in Periostin (POSTN), a protein produced by fibroblasts, as a major putative player in human carcinogenesis [8]. During embryogenesis, this protein is preferentially expressed in the periosteum and periodontal ligaments where it acts as a critical regulator of bone and tooth formation and maintenance [9]. However, it was also shown to play an important role in cardiac development [10]. In adults, POSTN is up-regulated by mechanical stress and contributes to tissue repair and regeneration [11,12]. It has recently been suggested that POSTN might also play a relevant role in human carcinogenesis. In fact, this protein interacts with multiple cell-surface receptors, most notably integrins, as well as with signals mainly via the PI3-K/Akt and other pathways**,** thus promoting cancer cell survival, epithelial–mesenchymal transition (EMT), invasion, and metastasis [13]. Though it is currently not clear whether the production and secretion of POSTN is directly mediated by tumor epithelial cells or by stromal cells, or by both, overexpression of POSTN in cancer stroma and/or in the epithelium is usually associated with the most malignant phenotypes and with poor clinical outcome [8]. In bladder cancer however, protein down-regulation was shown to be associated with poorer prognostic features [14], suggesting that POSTN can act either as a tumor promoter or as a tumor suppressor gene, most likely depending on several variables, including the protein isoform and /or the interactor involved in the process.

To the best of our knowledge, to date only two studies have investigated the clinical relevance of POSTN overexpression in PCa [15,16]. In one study, increased epithelial expression was found during the early stages of PCa, whereas stromal POSTN expression prevailed in advanced stages [15]. In the other study, which also showed POSTN to be far more overexpressed in tumor tissues than in peritumoral tissues, POSTN appeared to be overexpressed both by the epithelial and by the stromal cells [16]. In this study, a strong association between epithelial expression and local tumor stage was observed, while stromal overexpression appeared to be correlated mainly with a high Gleason score and an increased risk of biochemical failure [16].

In the present study we investigated POSTN expression in PCa tissue specimens and in normal peritumoral specimens in order to confirm previous findings and to evaluate the putative prognostic value of POSTN also as a function of its compartmentalization.

## Methods

### Patient selection

Since we originally planned to study POSTN overexpression in cryopreserved material by immune blotting techniques, a patient cohort we had previously utilized for studies on NM proteins was selected [7]. This cohort is made up of 90 patients who underwent radical prostatectomy for biopsy proven PCa between October 1995 and October 2003, and who were subsequently referred to our Unit for treatment or follow-up. Before surgery, all patients had provided consent allowing tumor tissue specimens to be collected for proteomic analysis. This research project was approved by the Ethical Committee of the National Cancer Research Institute of Genoa, Italy. Unfortunately, most of the stored material had been used for previous studies on NM proteins and therefore large enough samples for the present evaluations were no longer available. This prompted us to retrieve corresponding archival material in order to allow us to carry out immune histochemical studies. We were able to follow-up most of our cohort patients at regular intervals. However, over time, a relevant number of patients failed to attend clinical examinations**,** so the vital status of these patients had to be checked by phone, or, when this was not possible, by contacting the local tumor registry or the registry office of the patient’s place of residence. The main characteristics of the patients making up the study cohort are summarized in Table 1.

**Table 1:** Main characteristic of study patients

	**Total patients**
	**N=90(%)**
**Median age in years(range)**	64(48–77)
**Median PSA level at surgery in ng/ml(range)**	11.0(1.7-167.4)
**Extra-prostatic extension**	46(51.1)
**Pelvic nodes involved**	16(17.8)
**Surgical margins involved**	34(37.8)
**Seminal vesicles involved**	24(26.7)
**Gleason score<7**	32(35.6)
**=7**	30(33.3)
**>7**	28(31.1)

### Immunohistochemistry (IHC)

IHC analysis was carried out using 3-μm sections of paraffin embedded prostate tissue using the POSTN (OSF-2) Polyclonal Antibody (Acris Antibodies, Germany; Host/Isotype:Rabbit), suitable for various isoforms of POSTN. The antibody was diluted at 1:500.

The most representative tumor and normal peritumoral tissue sections were immunostained using the Benchmark XT automatic stainer (Ventana Medical Systems, SA Stasbourg, France). Slides were deparaffinized**,** and after adding high Ph, heat induced, standard citrate buffer (30 min), the antibody-antigen complex was relieved using the polymeric detection system (Ventana Medical System Ultraview Universal DAB Detection Kit). A negative and a positive control were used for each staining run. The negative control consisted of performing the entire IHC procedure on adjacent sections in the absence of the primary antibody. Then the sections were counter-stained with Gill's modified hematoxylin, cover-slipped and evaluated by two different observers using an Olympus multi-headed light microscope using 10X, 40X and 63X magnifications**.**

### Evaluation of staining

**Figure 1:**
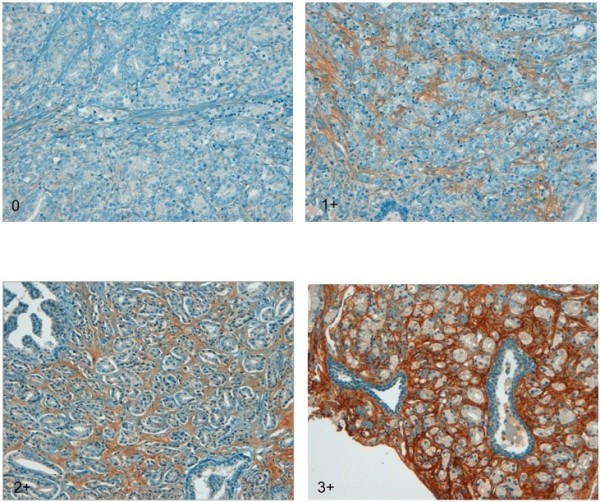
POSTN expression in tumor stroma: tumor specimens graded 0 to 3+ according to arbitrary scoring (see text) are shown.

**Figure 2:**
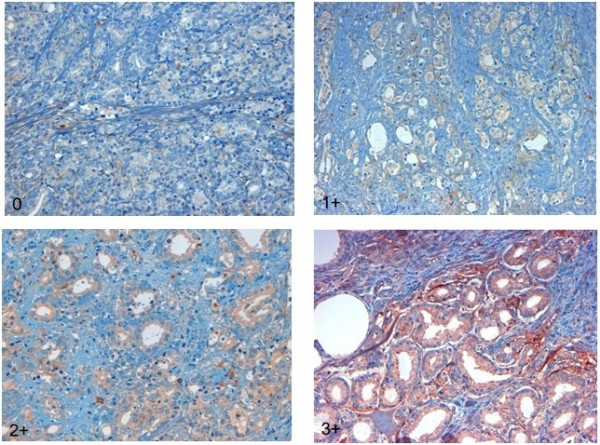
POSTN expression in tumor epithelium: tumor specimens graded 0 to 3+ according to arbitrary scoring (see text) are shown.

To evaluate epithelial and stromal POSTN expression, we used the immuno-reactive score (IRS) as previously implemented by Tischler et al. [16], based on the intensity of immune staining and the quantity of stained cells. The intensity of staining was arbitrarily graded as: absent (0), weak (1+), moderate (2+), strong (3+)**.** The percentage of stained cells was used to quantify the reaction as negative (0% of positive cells), 1+ (<10% positive cells); 2+ (10-50% of positive cells); 3+ (51-80% of positive cells); 4+ (>80% of positive cells). The final value of the analysis of each tissue sample was then expressed as an absolute value through the obtained score by multiplying the two individual scores (i.e., intensity of staining score times the percentage of stained cells score). Examples of scoring according to staining intensity and the percentage of stained cells are shown in Figures 1 and 2.

### Main analysis endpoints

PSA progression-free survival and overall survival were the main end-points of the present analysis. PSA progression was defined by any PSA serum level of 0.4 ng/ml or more following prostatectomy, provided that this value had been confirmed at least once, and at least 4 weeks apart. PSA progression-free survival was thus defined by the amount of time that elapsed from the date the patient underwent prostatectomy to the date of the documented PSA progression as defined above. Overall survival was calculated as the amount of time between the date of prostatectomy and the date of death, regardless of the cause.

### Statistical analysis

The t-test was applied to compare mean values (Standard Error: SE) of stromal or epithelial IRS in PCa tissue specimens with those calculated in normal peritumoral tissue specimens. Pearson’s correlation was used to correlate either epithelial or stromal POSTN expression with all of the following: PSA, Gleason score, extra-prostatic extension, lymph node status, involvement of surgical margins or of seminal vesicles. PSA progression-free and overall survival curves were constructed by the Kaplan–Meier method [17] and compared using the log-rank test [18]. To evaluate the role of prognostic variables, a series of Cox proportional hazards models were fitted to PSA progression-free and overall survival data [19]. The following covariates were included in all models: pre-surgery PSA levels (<=10 ng/ml, >10  ng/ml); extra-prostatic extension (Yes, No); involvement of surgical margins (No, Yes); involvement of seminal vesicles (No, Yes); Gleason score (Model I: <7, >=7), (Model II: <=7, >7), (Model III: <7, =7, >7); involvement of pelvic nodes (N0, N+); epithelial IRS; stromal IRS and stromal plus epithelial IRS. Relative to IRS, the value corresponding to the 75th percentile was used as an arbitrary cut-off (75th percentile value of epithelial IRS=2; 75th percentile value of stromal IRS=12). The 75th percentile value was infact the value which better discriminated the patient-cohort according to the main clinical outcome endpoints on study.

A stepwise procedure was used with a significance level of p=0.05 to retain variables in the model. Hazard Ratio (HR) estimates and their 95% Confidence Intervals (CIs) were also calculated [18]. All P values were two-tailed. The IBM software Statistical Package for Social Sciences (SPSS) version 19.0 for Windows (SPSS Inc. Chicago, Illinois, USA) was used for data analysis.

## Results

### POSTN expression in the epithelium and stroma of prostate tissues

Distinct stromal and epithelial staining characteristics allowed for absolutely certain evaluation of POSTN staining in the selected tumor and peritumor tissue specimens. Of the 90 prostate cancers, 79 (87.8%) displayed moderate (N°=32) or strong (N°=47) stromal POSTN expression. The mean (SE) overall IRS value was 6.37 (0.39). The expression of POSTN by tumor epithelial cells was significantly lower than what was observed in stromal cells (p=0.003). In fact, only 36 out of 90 (40%) cancer specimens showed weak (N°=25) or moderate (N°=11) staining intensity. The mean (ES) overall IRS in this case was 1.24 (0.21). Notably, stromal POSTN expression significantly correlated with epithelial POSTN expression (p<0.000). As shown in Table 2, both stromal and epithelial POSTN expressions were significantly increased in tumor tissues as compared to normal adjacent tissues (p<0.000 and p=0.001, respectively). Nevertheless, POSTN expression in the stromal component of normal tissues was about twice as high as what was observed in the epithelial component of prostate cancer tissues (p=0.003), indicating that stromal cells mostly contribute to POSTN secretion both in normal and in neoplastic conditions.

**Table 2:** POSTN expression in epithelium or stroma of tumor or normal tissue specimens

	**Epithelium**		**Stroma**	
	**Mean value(SE)**	**p≤**	**Mean value(SE)**	**p≤**
**Tumor tissue:Intensity**	0.56(0.08)		2.38(0.08)	
**Normal tissue:Intensity**	0.26(0.05)	0.002	1.49(0.09)	0.000
**Tumor tissue:%Cells stained**	0.89(0.13)		2.46(0.11)	
**Normal tissue:%Cells stained**	0.41(0.09)	0.003	1.52(0.10)	0.000
**Tumor tissue:IRS**	1.24(0.21)		6.37(0.39)	
**Normal tissue:IRS**	0.46(0.10)	0.001	2.80(0.27)	0.000

Abbreviations:SE,Standard Error;IRS, Immuno-reactive score.

### Correlation with clinical-pathological variables

We found no specific correlation between epithelial or stromal POSTN expression and any of the clinical-pathological parameters (PSA preoperative level; Gleason score; extra-prostatic extension; lymph node status; involvement of surgical margins or seminal vesicles) that we evaluated in the present analysis except for a weak positive correlation between POSTN stromal expression and Gleason score (p=0.08), and a significant correlation between epithelial expression of the protein and extra-prostatic extension (p=0.03).

### Correlation of POSTN expression with PSA-free and overall survival

**Figure 3:**
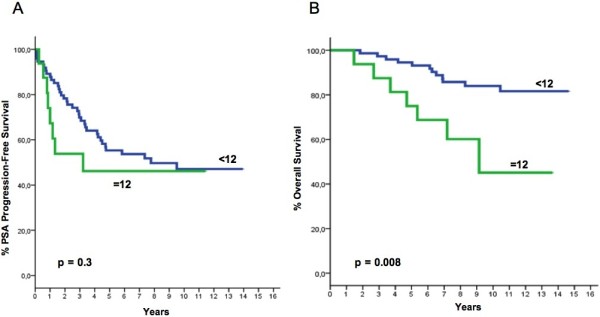
PSA progression-free Survival (A) and Overall Survival (B) as a function of stromal IRS (see text for explanations).

**Figure 4:**
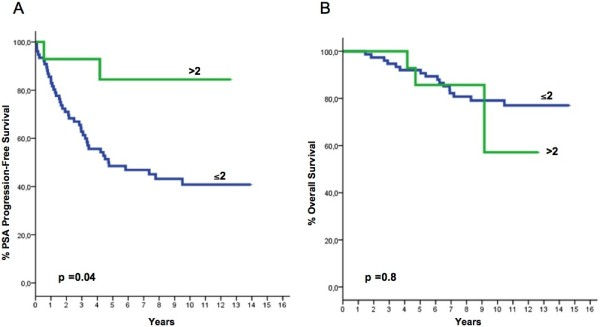
PSA progression-free (A) and Overall Survival (B) as a function of epithelial IRS (see text for explanations).

**Figure 5:**
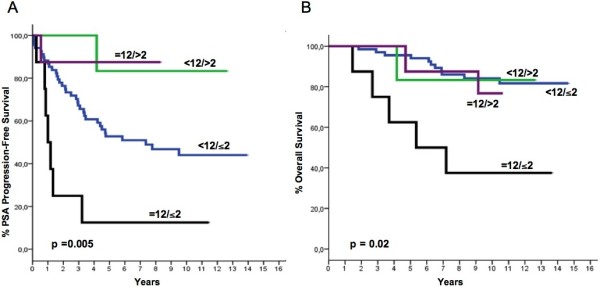
PSA progression-free (A) and Overall Survival (B) as a function of both stromal and epithelial IRS (see text for explanations).

After a median follow-up time of 134.5 months (range, 33.7–178.2), 44 patients progressed and 19 died. Median time to PSA progression for the whole cohort was 94.5 months (range 3–169.6), while median time to death has not been reached yet. POSTN expression in the stromal and epithelial compartments of the tumor both correlated with PSA progression-free survival or overall survival, albeit in a different manner. Stromal POSTN expression was significantly associated with overall survival, for an IRS value of 12, corresponding to the 75th percentile. The group of patients with an IRS=12 (strong staining intensity and 80% of positive cells) showed significantly shorter survival than patients with an IRS<12 (p=0.008). These patients also showed a trend for shorter PSA-free survival (Figure 3). While no significant correlation was found between epithelial IRS and patients' survival, in contrast to previous findings, a higher epithelial score (IRS>2, again corresponding to the 75th percentile) was significantly correlated with longer PSA-free survival (p=0.04) (Figure 4). Since stromal and epithelial expression appeared to have different correlations despite being directly correlated to each other as previously mentioned, the two variables (i.e., stromal and epithelial POSTN scores) were both included in the same multiparametric models together with the other variables that predicted both PSA–free and overall survival in univariate analysis. Different models were created on the basis of the Gleason score (Tables 3 and 4). Multivariate analysis confirmed that epithelial expression independently correlated with the risk of PSA progression, regardless of how the Gleason score was analyzed. Low expression did in fact imply an increase in the risk of PSA failure, which became statistically significant when the Gleason score was analyzed by arbitrarily grouping patients with a score >7 with those showing a score equal to 7. Multivariate analysis also confirmed that stromal IRS did not predict the risk of PSA failure, regardless of which Gleason score variable was used. By contrast, stromal IRS was the only independent predictor of the risk of death, regardless of how the Gleason score variable was analyzed. Epithelial IRS was not predictive of the risk of death, similarly to all the other variables we considered, including extra-prostatic extension and Gleason score. However, the latter variables were predictors of the risk of PSA failure. Noteworthy, the patients showing both high stromal expression (IRS=12) and low epithelial expression (IRS<=2) made up a subgroup with a very bleak prognosis, showing both the shortest PSA-free (p=0.005) and the shortest overall survival (p=0.02) (Figure 5).This trend was confirmed by multivariate analysis (Tables 5 and 6).

**Table 3:** Multivariate analysis

**PSA Progression-Free Survival**									
		**Model 1**			**Model 2**			**Model 3**	
	**HR**	**(95%CI)**	**p=**	**HR**	**(95%CI)**	**p=**	**HR**	**(95%CI)**	**p=**
**PSA,ng/ml**									
**≤10ng/ml**	1.0			1.0			1.0		
**>10ng/ml**	1.41	(0.67-2.97)	0.4	1.26	(0.59-2.68)	0.5	1.30	(0.61-2.79)	0.5
**Extra-prostatic extension**									
**Yes**	1.0			1.0			1.0		
**No**	3.33	(1.31-8.49)	0.01	3.21	(1.28-8.07)	0.01	3.21	(1.27-8.09)	0.01
**Pelvic nodes involved**									
**N0**	1.0			1.0			1.0		
**N+**	1.56	(0.69-3.56)	0.3	1.38	(0.60-3.14)	0.4	1.40	(0.61-3.18)	0.4
**Surgical margins involved**									
**No**	1.0			1.0			1.0		
**Yes**	0.75	(0.31-1.79)	0.5	0.72	(0.31-1.67)	0.4	0.68	(0.29-1.62)	0.4
**Seminal vesicles involved**									
**No**	1.0			1.0			1.0		
**Yes**	0.77	(0.35-1.68)	0.5	0.64	(0.28-1.43)	0.3	0.64	(0.28-1.42)	0.3
**Gleason score**									
**<7**	1.0								
**≥7**	2.03	(0.91-4.55)	0.08						
**Gleason score**									
**≤7**				1.0					
**>7**				3.29	(1.63-6.65)	0.001			
**Gleason score**									
**<7**							1.0		0.003
**=7**							1.33	(0.54-3.26)	0.5
**>7**							3.90	(1.59-9.55)	0.003
**Epithelial IRS**									
**>2**	1.0			1.0			1.0		
**≤2**	6.20	(1.30-29.44)	0.02	4.85	(0.98-24.09)	0.05	4.86	(0.98-24.05)	0.05
**Stroma IRS**									
**<12**	1.0			1.0			1.0		
**=12**	2.06	(0.87-4.88)	0.1	1.90	(0.77-4.65)	0.1	1.84	(0.76-4.50)	0.6

Abbreviations:HR, Hazard Ratio;CI,Confidence Interval;PSA,Prostate-specific antigen;IRS,Immuno-reactive score.

**Table 4:** Multivariate analysis

**Overall survival**									
		**Model 1**			**Model 2**			**Model 3**	
	**HR**	**(95%CI)**	**p=**	**HR**	**(95%CI)**	**p=**	**HR**	**(95%CI)**	**p=**
**PSA,ng/ml**									
**≤10ng/ml**	1.0			1.0			1.0		
**>10ng/ml**	0.54	(0.15-2.03)	0.4	0.48	(0.12-1.86)	0.3	0.48	(0.12-1.87)	0.3
**Extra-prostatic extension**									
**Yes**	1.0			1.0	(0.76-17.35)		1.0		
**No**	3.43	(0.73-16.12)	0.1	3.63		0.1	3.48	(0.73-16.54)	0.1
**Pelvic nodes involved**									
**N0**	1.0			1.0			1.0		
**N+**	1.71	(0.50-5.79)	0.4	1.44	(0.43-4.83)	0.5	1.40	(0.42-4.72)	0.6
**Surgical margins involved**									
**No**	1.0			1.0			1.0		
**Yes**	1.37	(0.45-4.21)	0.6	1.38	(0.47-4.01)	0.6	1.33	(0.45-3.92)	0.6
**Seminal vesicles involved**									
**No**	1.0			1.0			1.0		
**Yes**	1.39	(0.49-3.94)	0.5	1.08	(0.37-3.10)	0.9	1.11	(0.38-3.25)	0.8
**Gleason score**									
**<7**	1.0								
**≥7**	2.13	(0.55-8.19)	0.3						
**Gleason score**									
**≤7**				1.0					
**>7**				2.80	(0.93-8.47)	0.07			
**Gleason score**									
**<7**							1.0		0.1
**=7**							1.43	(0.32-6.40)	0.6
**>7**							3.52	(0.79-15.61)	0.1
**Epithelial IRS**									
**>2**	1.0			1.0			1.0		
**≤2**	3.22	(0.56-18.48)	0.2	3.09	(0.51-18.63)	0.2	3.19	(0.52-19.47)	0.2
**Stroma IRS**									
**<12**	1.0			1.0			1.0		
**=12**	5.85	(1.82-18.77)	0.003	5.76	(1.84-18.07)	0.003	5.99	(1.87-19.22)	0.003

Abbreviations:HR,Hazard Ratio;CI Confidence Interval;PSA,Prostate-specific antigen;IRS Immuno-reactive score.

**Table 5:** Multivariate analysis

**PSA Progression-Free Survival**									
		**Model 1**			**Model 2**			**Model 3**	
	**HR**	**(95%CI)**	**p=**	**HR**	**(95%CI)**	**p=**	**HR**	**(95%CI)**	**p=**
**PSA,ng/ml**									
**≤10ng/ml**	1.0			1.0			1.0		
**>10ng/ml**	1.39	(0.66-2.96)	0.4	1.25	(0.58-2.68)	0.6	1.29	(0.60-2.78)	0.5
**Extra-prostatic extension**									
**Yes**	1.0			1.0			1.0		
**No**	3.30	(1.29-8.47)	0.01	3.21	(1.27-8.07)	0.01	3.20	(1.27-8.09)	0.01
**Pelvic nodes involved**									
**N0**	1.0			1.0			1.0		
**N+**	1.55	(0.68-3.55)	0.3	1.37	(0.60-3.14)	0.4	1.39	(0.61-3.18)	0.4
**Surgical margins involved**									
**No**	1.0			1.0			1.0		
**Yes**	0.75	(0.31-1.81)	0.5	0.72	(0.31-1.67)	0.4	0.68	(0.29-1.62)	0.4
**Seminal vesicles involved**									
**No**	1.0			1.0			1.0		
**Yes**	0.77	(0.35-1.70)	0.5	0.64	(0.28-1.44)	0.3	0.64	(0.28-1.43)	0.3
**Gleason score**									
**<7**	1.0								
**≥7**	2.02	(0.90-4.54)	0.09						
**Gleason score**									
**≤7**				1.0					
**>7**				3.29	(1.62-6.65)	0.001			
**Gleason score**									
**<7**							1.0		0.03
**=7**							1.32	(0.54-3.26)	0.5
**>7**							3.89	(1.58-9.54)	0.003
**Stromal -Epithelial IRS Score**									
**=12/≤2**	1.0		0.08	1.0		0.2	1.0		0.2
**<12/≤2**	0.47	(0.19-1.17)	0.1	0.52	(0.20-1.33)	0.2	0.53	(0.21-1.37)	0.2
**=12/>2**	0.14	(0.01-1.33)	0.09	0.19	(0.02-1.89)	0.2	0.19	(0.02-1.89)	0.2
**<12/>2**	0.09	(0.01-0.77)	0.03	0.11	(0.01-1.10)	0.06	0.12	(0.01-1.11)	0.06

Abbreviations:HR,Hazard Ratio;CI ,Confidence Interval; PSA,Prostate-specific antigen;IRS, Immuno-reactive score.

**Table 6:** Multivariate analysis

**Overall survival**									
		**Model 1**			**Model 2**			**Model 3**	
	**HR**	**(95%CI)**	**p=**	**HR**	**(95%CI)**	**p=**	**HR**	**(95%CI)**	**p=**
**PSA,ng/ml**									
**≤10ng/ml**	1.0			1.0			1.0		
**>10ng/ml**	0.49	(0.13-1.83)	0.3	0.45	(0.12-1.74)	0.2	0.45	(0.12-1.74)	0.2
**Extra-prostatic extension**									
**Yes**	1.0			1.0			1.0		
**No**	3.34	(0.71-15.66)	0.1	3.75	(0.79-17.88)	0.1	3.52	(0.74-16.77)	0.1
**Pelvic nodes involved**									
**N0**	1.0			1.0			1.0		
**N+**	1.72	(0.50-5.88)	0.4	1.45	(0.43-4.93)	0.5	1.41	(0.41-4.79)	0.6
**Surgical margins involved**									
**No**	1.0			1.0			1.0		
**Yes**	1.42	(0.46-4.38)	0.5	1.36	(0.46-3.99)	0.6	1.31	(0.44-3.89)	0.6
**Seminal vesicles involved**									
**No**	1.0			1.0			1.0		
**Yes**	1.41	(0.50-3.97)	0.5	1.06	(0.37-3.05)	0.9	1.12	(0.38-3.20)	0.8
**Gleason score**									
**<7**	1.0								
**≥7**	2.19	(0.56-8.62)	0.3						
**Gleason score**									
**≤7**				1.0					
**>7**				2.75	(0.90-8.42)	0.08			
**Gleason score**									
**<7**							1.0		0.2
**=7**							1.49	(0.33-6.81)	0.6
**>7**							3.56	(0.78-16.20)	0.1
**Stromal -Epithelial IRS Score**									
**=12/≤2**	1.0		0.01	1.0		0.01	1.0		0.01
**<12/≤2**	0.13	(0.04-0.44)	0.001	0.14	(0.04-0.46)	0.001	0.14	(0.04-0.45)	0.001
**=12/>2**	0.19	(0.02-1.37)	0.1	0.21	(0.03-1.58)	0.1	0.20	(0.02-1.53)	0.1
**<12/>2**	0.12	(0.01-1.32)	0.08	0.12	(0.01-1.43)	0.09	0.11	(0.01-1.37)	0.09

Abbreviations:HR,Hazard Ratio;CI ,Confidence Interval; PSA, Prostate-specific antigen;IRS,Immuno-reactive score.

## Discussion

Comparably to previous findings by Tischler et al. [16], we have demonstrated that POSTN is far more highly expressed in cancer tissues than in normal tissues. In our study, POSTN appears to be expressed mainly in the stromal compartment, both in normal and in cancerous tissues. In Tischler’s study, epithelial expression was higher in normal tissues, while in cancerous tissues it was higher in the larger test cohort, but it was lower in the smaller training cohort [16]. Tischler's findings on normal prostate gland tissues are comparable with those previously reported by Tsunoda et al. [15] who also found higher POSTN expression in normal epithelial cells. However, in this study, POSTN expression was also higher in the epithelium than in the stroma of cancerous tissues [15]. It is not easy to explain the differences that were observed in the three studies. Besides the number of patients and disease stage, these studies differ in some methodological aspects. In our own, as well as in Tischler’s study [16], POSTN detection was performed using the same rabbit polyclonal antibody capable of recognizing all the different POSTN isoforms. A polyclonal anti-POSTN antibody was also used by the Japanese investigators for their IHC determinations [15]. However, there were major differences concerning staining evaluation. In fact, we and Tischler used an immune score (IRS) obtained by multiplying the intensity of staining by the percentage of stained cells. Notably, comparable median IRS values were obtained by us and by the Swiss colleagues. However, the results obtained in our two studies are not comparable since patients were analyzed after arbitrarily grouping them in different manners. In fact, in the Swiss study, median IRS values were used as cut-off points to dichotomize the tumors into a “POSTN low” and “POSTN high” population, while we found that the cut off score that best defined patient risk was the 75th percentile. In Tsunoda's study [15], IHC analysis only took into consideration the quantitative expression of POSTN (positive: at least >5% of staining cells), without evaluating staining intensity.

The differences in how patients were grouped, i.e. according to their IRS, may have been particularly relevant when POSTN expression was correlated with clinical outcome.

Tischler’s study [16] evaluated the correlation between POSTN expression and PSA relapse- free survival. They showed that higher stromal POSTN was significantly associated with shorter PSA-free survival both in the training cohort and in the test cohort set. However, the difference between low and high POSTN subgroups was statistically significant only in the training set. This might reflect the different size of the two cohorts and/or the length of follow-up (45 vs 72 months, respectively). However, it might also be associated with the different characteristics of the two study populations. In fact, 46% of the patients making up the training set showed biochemical failure compared with 20% of those forming the validation set. No relationship between PSA-free survival and epithelial POSTN expression was reported in this study. Unfortunately, these investigators did not explain the criteria that were adopted to define PSA progression, nor did they attempt to correlate POSTN expression with patients' survival.

We also observed a direct relationship between stromal POSTN IRS and PSA-free survival, however the difference was not statistically significant, exactly as reported by Tischler et al. [16] in their validation set which was much larger than our own set but showed a comparable clinical outcome. However, low epithelial POSTN expression was associated with shorter PSA-free survival in our study, and epithelial expression was not predictive of patient survival. By contrast, stromal expression was highly predictive of the risk of death, while it was only a weak predictor of PSA progression. These findings suggest that POSTN might play a different biological role in tumor progression, depending on its compartmentalization. In fact, while POSTN down-regulation in PCa epithelium appears to be correlated with extra-prostatic extension and biochemical failure, both of which represent early events in the natural history of the disease, POSTN overexpression in the stroma appears to be highly predictive of the risk of death, a late event that usually follows distant spreading and the loss of hormone dependency. This differential effect has been confirmed by multivariate analysis and suggests that it may be possible to identify different tumor phenotypes which are characterized by an increasing risk of PSA failure and death. In this regard, it is certainly intriguing that the prognostic value of POSTN overexpression in stroma is especially evident in patients whose tumors down-regulate POSTN expression in the epithelial compartment, but not in those whose tumors also overexpress the protein in the epithelial component (Figure 5). The phenotype characterized by low POSTN expression in the epithelium and high protein expression in the stroma showed a bleak prognosis, both in terms of PSA-free and overall survival.

Moreover, multi-parametric models showed that the proteomic signature based on the epithelial and stromal expression of POSTN indeed added to the prognostic information provided by the currently available variables, including the Gleason score. It should be stated in this regard that the old Gleason grading system was used and that caution is therefore warranted in data interpretation. 

## Conclusions

Our findings should be regarded as merely exploratory and, as such, they should be evaluated with caution. Nonetheless, they warrant IHC methodological standardization and further validation of the potential usefulness of POSTN as a prognostic marker in larger prospective series.

## Competing interest

The authors declare no competing interest.

## Authors’ contributions

NPV participated in the design of the study, in data interpretation and in drafting the manuscript. RA participated in the design of the study and performed the statistical analysis. ZL made substantial contributions in updating patients histories and helped in statistical analysis. RF participated in study conception and acquisition of data. SS carried out the immunohistochemistry analysis and evaluation of staining. BS carried out the immunohistochemistry analysis and evaluation of staining. BF: made substantial contributions to conception, design, analysis and interpretation of data. He has been involved in drafting the manuscript and revising it critically for important intellectual content. He gave final approval of the version to be published. No degree of relationship do exhist between Boccardo S and Boccardo F, as they simply share an homonymy of family name. All authors read and approved the final manuscript.

## Pre-publication history

The pre-publication history for this paper can be accessed here:

http://www.biomedcentral.com/1471-2407/12/625/prepub
